# Transcriptomic profiling of 39 commonly-used neuroblastoma cell lines

**DOI:** 10.1038/sdata.2017.33

**Published:** 2017-03-28

**Authors:** Jo Lynne Harenza, Maura A. Diamond, Rebecca N. Adams, Michael M. Song, Heather L. Davidson, Lori S. Hart, Maiah H. Dent, Paolo Fortina, C. Patrick Reynolds, John M. Maris

**Affiliations:** 1 Division of Oncology and Center for Childhood Cancer Research, Children’s Hospital of Philadelphia, Philadelphia, Pennsylvania 19104, USA; 2 Cancer Genomics and Bioinformatics Laboratory, Sidney Kimmel Cancer Center, Philadelphia, Pennsylvania 19107, USA; 3 Cancer Center, Texas Tech University Health Sciences Center School of Medicine, Lubbock, Texas 79430, USA; 4 Department of Pediatrics, Perelman School of Medicine at the University of Pennsylvania, Philadelphia, Pennsylvania 19104, USA

**Keywords:** Paediatric cancer, Cancer genomics, RNA sequencing, Gene expression analysis

## Abstract

Neuroblastoma cell lines are an important and cost-effective model used to study oncogenic drivers of the disease. While many of these cell lines have been previously characterized with SNP, methylation, and/or mRNA expression microarrays, there has not been an effort to comprehensively sequence these cell lines. Here, we present raw whole transcriptome data generated by RNA sequencing of 39 commonly-used neuroblastoma cell lines. These data can be used to perform differential expression analysis based on a genetic aberration or phenotype in neuroblastoma (e.g., *MYCN* amplification status, *ALK* mutation status, chromosome arm 1p, 11q and/or 17q status, sensitivity to pharmacologic perturbation). Additionally, we designed this experiment to enable structural variant and/or long-noncoding RNA analysis across these cell lines. Finally, as more DNase/ATAC and histone/transcription factor ChIP sequencing is performed in these cell lines, our RNA-Seq data will be an important complement to inform transcriptional targets as well as regulatory (enhancer or repressor) elements in neuroblastoma.

## Background & Summary

An estimated 15,780 children were diagnosed with cancer in 2014 In the United States, and per year globally, this number is nearly 250,000 (ref. 1). Although the 5-year survival rate of pediatric cancers is ~80%, many of the most commonly diagnosed childhood cancers: brain tumors, Wilms tumor, rhabdomyosarcoma, and high-risk neuroblastoma, have devastatingly low rates of survival^1^, demonstrating the continued need for research progress in these areas. Here, we focus on neuroblastoma, the most common extracranial solid tumor in children. This disease has an estimated incidence of 1 in 8,000 to 10,000 births^2^ and a 5-year survival rate of >95% for children in the low and intermediate risk groups. However, children with high-risk disease have only a 40% likelihood of survival^2^. Culturing of neuroblastoma cell lines dates back to the 1940s (ref. 3), during which the sole purpose of culturing was for diagnosis. However, producing cell lines from neuroblastoma tumors quickly became routine (see review^4^) and today, they are commonly-used, highly-characterized models used in laboratories across the world. Neuroblastoma cell lines nicely model a tumor’s histopathology, gene expression, aneuploidy, and drug sensitivity, thus they are routinely used to investigate oncogenes or signaling pathways pharmacologically (drug screens, drug sensitivity/resistance) and/or genetically (siRNA, shRNA, CRISPR).

The genomics of neuroblastoma cell lines have been previously characterized using SNP^5^, methylation^5,6^, and/or mRNA expression microarrays^7–9^, however, there has not been an effort to profile a large panel of these cell lines with high-throughput sequencing techniques. The motivation behind this study was to comprehensively profile the mRNA and non-coding RNA transcriptome of commonly-used neuroblastoma cell line models with a major goal of using this information as a complement to the epigenomic data currently available and the many data in the process of being generated. Integration of RNA expression patterns with histone and/or transcription factor chromatin immunoprecipitation (ChIP) sequencing is necessary for inferring transcriptional regulatory events. Neuroblastomas can be classified into various groups based on genetic lesions, for example: MYCN copy number amplification, harboring an activating *ALK* mutation, harboring a chromosomal loss (e.g.,: 1p, 3p, 11q) or gain (17q), *TERT* rearrangements (for review of neuroblastoma genomics, see ref. 10). Utilizing a panel of cell lines which harbor a mixture of these characteristics enables differential expression analyses on the basis of a genetic lesion, mutation of interest, or expression of a gene of interest.

These data have reuse value to inform selection of cell lines for experimental investigation of putative neuroblastoma oncogenes and/or tumor suppressors. For example, choice of knock-down or over-expression studies require *a priori* knowledge of basal expression of the gene of interest for rational experimental design. These data allow the experimenter to quickly determine which cell lines are high, mid, or low expressers of a gene of interest without requiring tedious quantitative, real-time PCR analysis or western blotting of multiple cell lines prior to initiating a gene over-expression or knockdown experiment.

**Figure 1:**
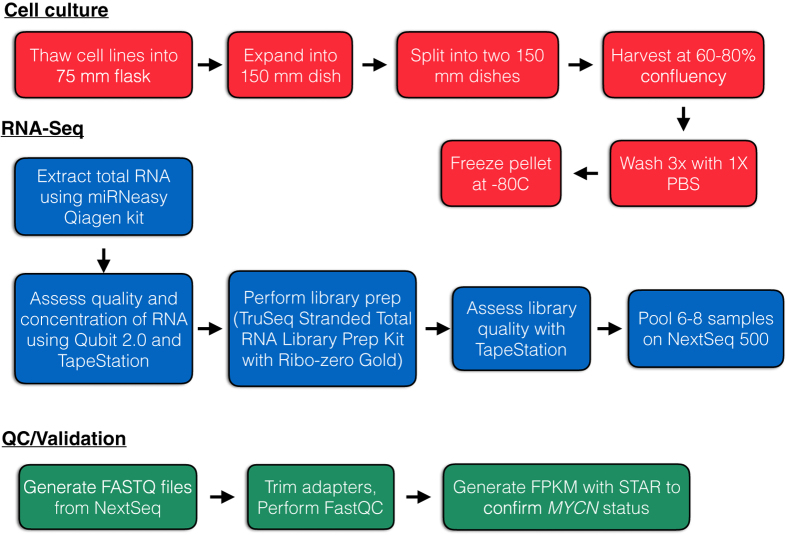
Experimental and data analysis workflow. Cell lines were thawed and cultured to ~60–80% confluence before passaging and finally, pelleting. RNA was extracted, sequencing performed, and data analysis performed as described.

Here, we describe transcriptome-wide profiling of 39 neuroblastoma cell lines, the hTERT-immortalized retinal pigmented epithelial cell line, RPE-1, and pooled human fetal brain tissue. Careful and stringent technical design at each experimental stage has allowed generation of a high-quality RNA-Seq dataset which has tremendous reuse value for the neuroblastoma community. An overview of the study design is depicted in Fig. 1. Briefly, cell lines were thawed, grown, and collected at 60–80% confluency over a two-month period. Once all cell lines were pelleted, RNA extractions were performed, quality of RNA inspected, and RNA sequencing was performed. Raw FASTQ files were generated and are publicly-available for reuse (see Data Citation 1). Additionally, we provide a processed file of gene-level mRNA abundances for each sample. We anticipate this data being a valuable tool for the neuroblastoma research community as we continue investigation into oncogenomic mechanisms of this disease.

## Methods

### Cell lines and culturing

Cell line stocks were obtained from the Children’s Oncology Group (COG) Cell Culture and Xenograft Repository at Texas Tech University Health Sciences Center (www.COGcell.org), the American Type Culture Collection (Manassas, VA), or the Children’s Hospital of Philadelphia (CHOP) cell line bank. Several of the COG-derived cell lines were established direct-to-culture in parallel with a patient-derived xenograft model^11^ that are being characterized separately (see Table 1 (available online only)). All cell culturing for this experiment was performed at CHOP. Each cell line was thawed for 2–3 min in a 37 °C water bath, added to a 15 ml tube containing its respective growth medium, and pelleted by centrifugation at 300×g for 3 min. The supernatant was discarded to remove the DMSO-containing freezing medium. Cells were re-suspended in 1 ml of growth medium and transferred into a T75 flask containing an additional 10 ml of growth medium. Once cells were ~70–80% confluent, they were transferred to a 150 mm dish. At ~70–80% confluency, cells were split into two 150 mm dishes and at ~70–80% confluency, each dish of cells was pelleted, washed 3x with 1X PBS, and frozen at −80C until nucleic acids were extracted. See Table 1 (available online only) for a complete listing of cell lines, whether a matched patient-derived xenograft (PDX) exists, and their growth medium. Cell lines appended with ‘nb’ were grown in serum-free neurobasal medium. The following were purchased from Thermo Fisher Scientific (Waltham, MA): Iscove’s IMDM (Cat# 12440053), RPMI 1640 with 25 mM HEPES (Cat# 22400089), Neurobasal-A Medium (Cat# 10888022), L-glutamine (Cat# 25030081), antibiotic/antimycotic (Cat# 15240062), 50X B-27 serum-free supplement (Cat# 18504044), 100X N-2 supplement (Cat# 17502048). The following growth factors were purchased from VWR (Radnor, PA): rhFGF (fibroblast growth factor, Cat# PAG5071) and rhEGF (epidermal growth factor, Cat# PAG5021). Insulin/Transferrin/Selenium (ITS) premix culture supplement was purchased from Corning Life Sciences (Tewksbury, MA, Cat# 354351). Hyclone Fetal bovine serum was purchased from Fisher Scientific (Cat# SH30071.03) and the lot remained consistent across the different medium formulations throughout the duration of the experiment. Of note, SK-N-BE(2)-C is a subclone derived from the parental SK-N-BE(2) cell line^12^ and SH-SY5Y was derived from the SH-SY subclone of the parental SK-N-SH cell line^13^.

Throughout the duration of the study, randomization was implemented to ensure unbiased data production. Cell lines were thawed in random order, nucleic acid extractions were performed randomly, and library preps and sequencing were performed randomly. Phenotypic characteristics of each cell line were also assessed as quality control during the cell growth stage. No unusual morphologies or growth rates were noted.

### DNA extraction and STR profiling

From separate cell pellets, DNA was extracted using the DNeasy Blood & Tissue Kit (Cat# 69504, Qiagen, Valencia, CA). DNA was quantitated using the Nanodrop 1000 (Thermo Fisher Scientific) and Short Tandem Repeat (STR) profiling employed either the AmpFLSTR Identifiler PCR Amplification kit (Applied Biosystems, Foster City, CA) by the Children’s Hospital of Philadelphia Nucleic Acids and Protein Core or the PowerPlex Fusion kit (Promega, Madison, WI) by Guardian Forensic Sciences (Abington, PA). All cell line STRs matched publicly-available references listed at http://strdb.cogcell.org/.

### RNA extraction

Control human fetal brain total RNA (Cat# 636526, Lot#1605061A) was purchased from Clontech Laboratories (Mountain View, CA). This RNA was a pool of normal brain tissue from 21 spontaneously aborted male/female Caucasian fetuses of ages 26–40 weeks and was isolated using a modified guanidinium thiocyanate method^14^. For all cell lines, RNA was extracted using the miRNeasy Mini kit (Cat# 217004) from Qiagen (Valencia, CA) according to the manufacturer’s protocol. RNA purity was assessed using the Nanodrop 2000 (Thermo Fisher Scientific) and quantitated with the Qubit 2.0 Fluorometer (Thermo Fisher Scientific). Quality and RNA integrity numbers (RINs) were assessed using the TapeStation 2200 (Agilent Technologies, Santa Clara, CA). Each cell line RNA sample had a RIN≥8.7 and the RIN for the fetal brain RNA was 7.6, thus all RNA was of high quality.

### Library preparation and RNA sequencing

Libraries were prepared using 1 ug RNA according to the TruSeq Stranded Total RNA Sample Preparation guide (Part# 15031048 Rev. E, October 2013, Illumina, San Diego, CA). Ribosomal RNA removal was performed using the Gold rRNA Removal Mix per Illumina's recommendations. Quality of each library assessed with the Agilent TapeStation 2,200. Six to eight libraries were pooled (*N*=6–8) and sequenced using v2 chemistry, 2×100 bp, on one high-output flow-cell of an Illumina NextSeq 500 to achieve at least 50 million paired reads per sample. Upon run completion, libraries were demultiplexed, Illumina adapters trimmed, and FASTQ files were generated using the Illumina NextSeq Control Software version 2.02.

### Sequencing quality control

First, sample reads were concatenated for each paired read group. Next, FASTQC V0.11.4 (Babraham Institute, available for download at http://www.bioinformatics.babraham.ac.uk/projects/fastqc/) was run on all samples and inspected for sequencing quality. Next, Picard tools version 1.140 (Broad Institute, Cambridge, MA, available for download at https://github.com/broadinstitute/picard/releases/tag/1.140) was used to calculate insert sizes for GEO according to the following parameters:

$ java -jar picard.jar CollectInsertSizeMetrics INPUT=Aligned.sortedByCoord.out.bam OUTPUT=filename

### Alignment and generation of counts

The Spliced Transcripts Alignment to Reference (STAR) version 2.4.2a aligner (available for download at https://github.com/alexdobin/STAR/releases/tag/STAR_2.4.2a)^15^ was used to index the full hg19 genome fasta file from UCSC using the following parameters:

$ STAR --runMode genomeGenerate --runThreadN 16 --genomeDir idx_dir --genomeFastaFiles ucsc.hg19.fa --sjdbGTFfile refSeq_hg19_2016-03-03.gtf --sjdbOverhang 100

The GTF file was downloaded using the **genePredToGtf** command from the kent utility (available for download at http://hgdownload.cse.ucsc.edu/admin/exe/linux.x86_64/):

$ genePredToGtf hg19 knownGene knownGene.gtf

Next, sequences were aligned and counts per gene were generated using the following parameters in two-pass mode:

$ STAR --runMode alignReads --runThreadN 16 --twopassMode Basic --twopass1readsN -1 --chimSegmentMin 15 --chimOutType WithinBAM --genomeDir dir --genomeFastaFiles ucsc.hg19.fa --readFilesIn R1.fastq.gz R2.fastq.gz --readFilesCommand zcat --outSAMtype BAM SortedByCoordinate --outFileNamePrefix $cellline. --quantMode TranscriptomeSAM GeneCounts --sjdbGTFfile refSeq_hg19_2016-03-03.gtf --sjdbOverhang 100

Alignment resulted in an average of 66 million uniquely-mapped reads per sample. STAR two-pass mode alignment was chosen as it has been shown to have 99% alignment accuracy and has nearly 20x faster processing speed compared with TopHat2 and similar processing speed as HISAT two-pass mode^16^.

### Generation of FPKM

A custom R script was used to generate gene fragments per kilobase of exons per million reads (FPKM) from the count data produced from STAR. The Genomic Features Package version 1.22.13 (available for download at https://bioconductor.org/packages/release/bioc/html/GenomicFeatures.html) was used with R Version 3.2.2 (Fire Safety) to make the transcriptome database and figures were produced using ggplot2 version 2.1.0 (http://ggplot2.org/).

### Differential expression analyses

Differential expression of genes based on *MYCN* amplification status was performed separately for cell lines and primary neuroblastoma tumor samples using the R package, DESeq2 (version 1.10.1)^17^. FASTQ files and *MYCN* status for patient tumors were obtained with consent through the Therapeutically Applicable Research to Generate Effective Treatments (TARGET) Consortium (see Data Citation 2, https://ocg.cancer.gov/programs/target/data-matrix). Next, the differentially-expressed genes’ log_2_-transformed mean expression and the log_2_ fold-change were correlated between the cell lines and patient samples.

### Code availability

R scripts for generation of FPKM and differential expression analyses are available for download at: https://github.com/marislab/NBL-cell-line-RNA-seq.

## Data Records

All raw RNA-sequencing data (paired FASTQ files) as well as the processed FPKM matrix from this study have been deposited into the Gene Expression Omnibus (GEO) under Accession Number GSE89413 (see Data Citation 1). For associated specimen metadata, see Table 1 (available online only) and for associated assay metadata, see Table 2 (available online only). Raw single nucleotide polymorphism (SNP) array IDAT files and processed Genome Studio files for 27 of the cell lines have been deposited into GEO under Accession Number GSE89968 (see Data Citation 3). Together, these data make up the GEO Super Series GSE89969.

## Technical Validation

**Figure 2:**
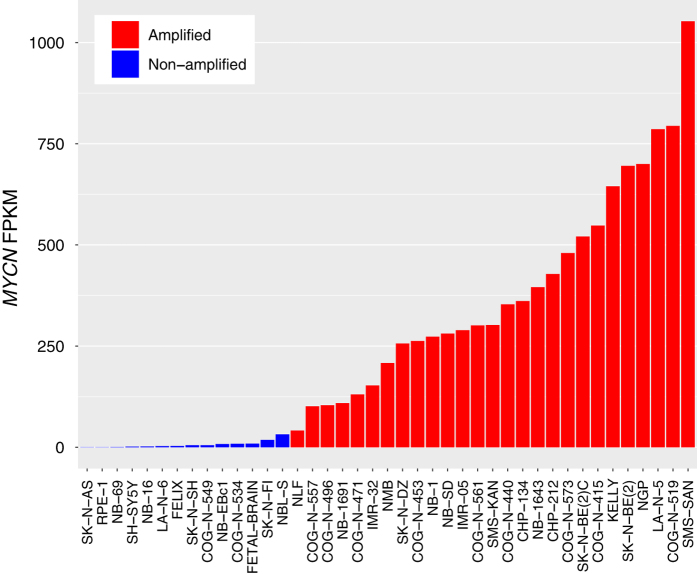
Validation of *MYCN* genomic amplification status in neuroblastoma cell lines. Plotted are rank-ordered *MYCN* FPKM values for the human fetal brain sample and each cell line, colored by known *MYCN* copy number status. These data validate known *MYCN* amplification status for each cell line.

As a technical validation of our RNA-Seq data, we generated FPKM for all genes (See Methods and Data Citation 1) and compared *MYCN* FPKM with each cell line’s known copy number amplification status across cell lines (Fig. 2 and Table 3 (available online only)) . Of note, the tumor from which the NLF cell line was derived was *MYCN* copy number amplified by the fluorescence *in situ* hybridization, however, it is not amplified at the protein level^18^ and therefore, as expected, has the lowest *MYCN* FPKM of all cell lines designated as *MYCN* amplified. All cell lines were concordant with known *MYCN* amplification status.

**Figure 3:**
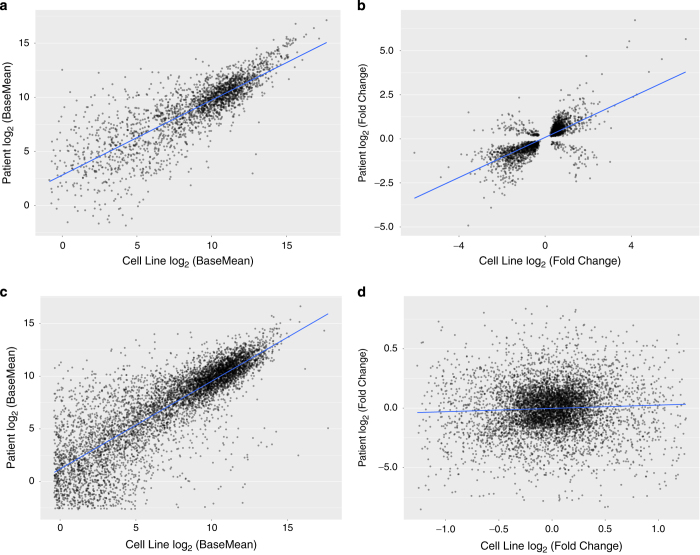
Concordance of differentially-expressed genes between neuroblastoma cell lines and primary tumors. (**a**) Across the neuroblastoma cell lines, 3,940 genes were differentially-expressed (DE) based on *MYCN* amplification status and of those, 2,395 were differentially-expressed based on *MYCN* amplification status in primary tumors and were significantly correlated (Pearson’s R=0.824, *P*<2.2 e-16). (**b**) The fold changes of these DE genes were significantly correlated between the cell line dataset and the patient tumor dataset (Pearson’s R=0.73, *P*<2.2 e-16). (**c**) A significant correlation between the common 6,523 genes that were not DE in cell lines and tumors was observed (Pearson’s R=0.829, *P*<2.2 e-16). (**d**) As expected, correlation of the non-DE genes’ fold changes was close to zero (Pearson’s R=0.052, *P*<3 e-5).

Next, for both cell lines and neuroblastoma patient data, we performed differential expression analyses based on *MYCN* genomic amplification status using the R package, DESeq2 (ref. 17). We correlated the DESeq2 base mean of the common differentially-expressed genes (*N*=2,395) between cell lines and primary patient tumors, which were significantly correlated (Fig. 3a, Pearson’s R=0.824, t=71.131, df=2,393, 95% CI=0.811–0.836, *P*<2.2 e-16). The fold changes of these genes were also significantly correlated between the cell lines and patient samples (Fig. 3b, Pearson’s R=0.73, t=52.231, df=2,393, 95% CI=0.711–0.748, *P*<2.2 e-16), not only supporting the technical validity of our dataset, but also emphasizing the utility of these cell lines as a surrogate model for neuroblastoma.

Finally, we correlated non-differentially expressed genes (DESeq2 p-adjusted > 0.20) between the cell lines and patient tumors (*N*=6,523). As expected, base mean expression of the genes correlated significantly (Fig. 3c, Pearson’s R=0.829, t=119.74, df=6,521, 95% CI=0.821–0.837, *P*<2.2 e-16). While correlating fold-change yields a significant *P*-value because of the large number of genes analyzed, it is clear that the relationship is weak, as the correlation is close to zero (Fig. 3d, Pearson’s R=0.052, t=4.1,766, df=6,521, 95% CI=0.027–0.076, *P*<3 e-5). This is expected, as all fold-changes of non-DE genes are close to zero.

## Usage Notes

All raw FASTQ files and the associated FPKM matrix file can be downloaded from the Gene Expression Omnibus (GEO) under Accession Number GSE89413. STAR-Fusion (https://github.com/STAR-Fusion/STAR-Fusion) enables detection of fusion transcripts. Alternative gene expression analyses can be performed using RSEM^19^ and/or transcript level analyses can be performed using kallisto^20^. Use of kallisto will also allow quantification of non-coding RNA abundances. Differential expression analyses may be performed using the common R packages, limma^21^ or DESeq2 (ref. 17). Differentially expressed gene lists can be explored for enrichment in signaling pathways using Ingenuity Pathway Analysis (Qiagen, http://www.ingenuity.com/products/ipa) and/or gene ontologies using ToppGene^22^ or the Gene Ontology Consortium tool^23^. Finally, these expression data can be integrated with epigenomics datasets (e.g.: ChIP-Seq, DNase-Seq/ATAC-Seq, Histone ChIP-Seq) to infer transcriptional regulation or repression.

## Additional Information

**How to cite this article:** Harenza, J. L. *et al.* Transcriptomic profiling of 39 commonly-used neuroblastoma cell lines. *Sci. Data* 4:170033 doi: 10.1038/sdata.2017.33 (2017).

**Publisher’s note:** Springer Nature remains neutral with regard to jurisdictional claims in published maps and institutional affiliations.

## Supplementary Material



## Figures and Tables

**Table 1:** Associated metadata for the cell lines used in this study

**Source Name**	**Characteristics [organism]**	**Characteristics [organism part]**	**Characteristics [Matched PDX]**	**Method**	**Sample Name**	**Factor Value [Growth Media]**	**Factor Value [Detachment Method]**
CHP-134 cell line	Homo sapiens	Neuroblastoma	NA	Total RNA extraction	CHP-134	RPMI 1,640, 10% FBS, 1% Penicillin/Streptomycin, 2 mM L-Glutamine	0.02% Versene
CHP-212 cell line	Homo sapiens	Neuroblastoma	NA	Total RNA extraction	CHP-212	RPMI 1,640, 10% FBS, 1% Penicillin/Streptomycin, 2 mM L-Glutamine	0.02% Versene
COG-N-415 cell line	Homo sapiens	Neuroblastoma	COG-N-415x	Total RNA extraction	COG-N-415	IMDM, 20% FBS, 1% Penicillin/Streptomycin, 2 mM L-glutamine, 1:1,000 ITS Premix Supplement	n/a
COG-N-440 cell line	Homo sapiens	Neuroblastoma	COG-N-440x	Total RNA extraction	COG-N-440	IMDM, 20% FBS, 1% Penicillin/Streptomycin, 2 mM L-glutamine, 1:1,000 ITS Premix Supplement	0.02% Versene
COG-N-453 cell line	Homo sapiens	Neuroblastoma	COG-N-453x	Total RNA extraction	COG-N-453	IMDM, 20% FBS, 1% Penicillin/Streptomycin, 2 mM L-glutamine, 1:1,000 ITS Premix Supplement	n/a
COG-N-471nb cell line	Homo sapiens	Neuroblastoma	COG-N-471x	Total RNA extraction	COG-N-471nb	Neurobasal-A plus 50 ng/ml rhEGF, 50 ng/ml rhFGF, 2 mM L-glutamine, 1X B27, and 1X N2	0.02% Versene
COG-N-496 cell line	Homo sapiens	Neuroblastoma	COG-N-496x	Total RNA extraction	COG-N-496	IMDM, 20% FBS, 1% Penicillin/Streptomycin, 2 mM L-glutamine, 1:1,000 ITS Premix Supplement	0.02% Versene
COG-N-519 cell line	Homo sapiens	Neuroblastoma	COG-N-519x	Total RNA extraction	COG-N-519	IMDM, 20% FBS, 1% Penicillin/Streptomycin, 2 mM L-glutamine, 1:1,000 ITS Premix Supplement	0.02% Versene
COG-N-534 cell line	Homo sapiens	Neuroblastoma	COG-N-534m	Total RNA extraction	COG-N-534	IMDM, 20% FBS, 1% Penicillin/Streptomycin, 2 mM L-glutamine, 1:1,000 ITS Premix Supplement	0.02% Versene
COG-N-549 cell line	Homo sapiens	Neuroblastoma	COG-N-549x	Total RNA extraction	COG-N-549	IMDM, 20% FBS, 1% Penicillin/Streptomycin, 2 mM L-glutamine, 1:1,000 ITS Premix Supplement	0.02% Versene
COG-N-557nb cell line	Homo sapiens	Neuroblastoma	COG-N-557x	Total RNA extraction	COG-N-557nb	Neurobasal-A plus 50 ng/ml rhEGF, 50 ng/ml rhFGF, 2 mM L-glutamine, 1X B27, and 1X N2	0.02% Versene
COG-N-561 cell line	Homo sapiens	Neuroblastoma	COG-N-561x	Total RNA extraction	COG-N-561	IMDM, 20% FBS, 1% Penicillin/Streptomycin, 2 mM L-glutamine, 1:1,000 ITS Premix Supplement	0.02% Versene
COG-N-573 cell line	Homo sapiens	Neuroblastoma	COG-N-573x	Total RNA extraction	COG-N-573	IMDM, 20% FBS, 1% Penicillin/Streptomycin, 2 mM L-glutamine, 1:1,000 ITS Premix Supplement	0.02% Versene
Felix cell line	Homo sapiens	Neuroblastoma	FELIXx	Total RNA extraction	Felix (COG-N-426)	IMDM, 20% FBS, 1% Penicillin/Streptomycin, 2 mM L-glutamine, 1:1,000 ITS Premix Supplement	0.02% Versene
IMR-05 cell line	Homo sapiens	Neuroblastoma	NA	Total RNA extraction	IMR-05	RPMI 1,640, 10% FBS, 1% Penicillin/Streptomycin, 2 mM L-Glutamine	0.02% Versene
IMR-32 cell line	Homo sapiens	Neuroblastoma	NA	Total RNA extraction	IMR-32	RPMI 1,640, 10% FBS, 1% Penicillin/Streptomycin, 2 mM L-Glutamine	0.02% Versene
KELLY cell line	Homo sapiens	Neuroblastoma	NA	Total RNA extraction	KELLY	RPMI 1,640, 10% FBS, 1% Penicillin/Streptomycin, 2 mM L-Glutamine	0.02% Versene
LA-N-5 cell line	Homo sapiens	Neuroblastoma	NA	Total RNA extraction	LA-N-5	RPMI 1,640, 10% FBS, 1% Penicillin/Streptomycin, 2 mM L-Glutamine	0.02% Versene
LA-N-6 cell line	Homo sapiens	Neuroblastoma	NA	Total RNA extraction	LA-N-6	RPMI 1,640, 10% FBS, 1% Penicillin/Streptomycin, 2 mM L-Glutamine	0.02% Versene
NB-1 cell line	Homo sapiens	Neuroblastoma	NA	Total RNA extraction	NB-1	RPMI 1,640, 10% FBS, 1% Penicillin/Streptomycin, 2 mM L-Glutamine	n/a
NB-16 cell line	Homo sapiens	Neuroblastoma	NA	Total RNA extraction	NB-16	RPMI 1,640, 10% FBS, 1% Penicillin/Streptomycin, 2 mM L-Glutamine	0.05% Trypsin/EDTA
NB-1,643 cell line	Homo sapiens	Neuroblastoma	NB-1,643	Total RNA extraction	NB-1,643	IMDM, 20% FBS, 1% Penicillin/Streptomycin, 2 mM L-glutamine	n/a
NB-1,691 cell line	Homo sapiens	Neuroblastoma	NB-1,691	Total RNA extraction	NB-1,691	RPMI 1,640, 10% FBS, 1% Penicillin/Streptomycin, 2 mM L-Glutamine	0.05% Trypsin/EDTA
NB-69 cell line	Homo sapiens	Neuroblastoma	NA	Total RNA extraction	NB-69	RPMI 1,640, 10% FBS, 1% Penicillin/Streptomycin, 2 mM L-Glutamine	0.02% Versene
NB-EBc1 cell line	Homo sapiens	Neuroblastoma	NB-EBc1	Total RNA extraction	NB-EBc1	RPMI 1,640, 10% FBS, 1% Penicillin/Streptomycin, 2 mM L-Glutamine	0.02% Versene
NB-SD cell line	Homo sapiens	Neuroblastoma	NB-SD	Total RNA extraction	NB-SD	RPMI 1,640, 10% FBS, 1% Penicillin/Streptomycin, 2 mM L-Glutamine	0.02% Versene
NBL-S cell line	Homo sapiens	Neuroblastoma	NA	Total RNA extraction	NBL-S	RPMI 1,640, 10% FBS, 1% Penicillin/Streptomycin, 2 mM L-Glutamine	0.02% Versene
NGP cell line	Homo sapiens	Neuroblastoma	NA	Total RNA extraction	NGP	RPMI 1,640, 10% FBS, 1% Penicillin/Streptomycin, 2 mM L-Glutamine	0.02% Versene
NLFcell line	Homo sapiens	Neuroblastoma	NA	Total RNA extraction	NLF	RPMI 1,640, 10% FBS, 1% Penicillin/Streptomycin, 2 mM L-Glutamine	0.02% Versene
NMB cell line	Homo sapiens	Neuroblastoma	NA	Total RNA extraction	NMB	RPMI 1,640, 10% FBS, 1% Penicillin/Streptomycin, 2 mM L-Glutamine	0.02% Versene
RPE-1 cell line	Homo sapiens	Epithelial	NA	Total RNA extraction	RPE-1	RPMI 1,640, 10% FBS, 1% Penicillin/Streptomycin, 2 mM L-Glutamine	0.05% Trypsin/EDTA
SH-SY5Y cell line	Homo sapiens	Neuroblastoma	NA	Total RNA extraction	SH-SY5Y	RPMI 1,640, 10% FBS, 1% Penicillin/Streptomycin, 2 mM L-Glutamine	0.02% Versene
SK-N-AS cell line	Homo sapiens	Neuroblastoma	SK-N-AS xenograft from cell line; not patient-derived	Total RNA extraction	SK-N-AS	RPMI 1,640, 10% FBS, 1% Penicillin/Streptomycin, 2 mM L-Glutamine	0.05% Trypsin/EDTA
SK-N-BE(2) cell line	Homo sapiens	Neuroblastoma	NA	Total RNA extraction	SK-N-BE(2)	RPMI 1,640, 10% FBS, 1% Penicillin/Streptomycin, 2 mM L-Glutamine	0.02% Versene
SK-N-BE(2)-C cell line	Homo sapiens	Neuroblastoma	NA	Total RNA extraction	SK-N-BE(2)-C	RPMI 1,640, 10% FBS, 1% Penicillin/Streptomycin, 2 mM L-Glutamine	0.02% Versene
SK-N-DZ cell line	Homo sapiens	Neuroblastoma	NA	Total RNA extraction	SK-N-DZ	RPMI 1,640, 10% FBS, 1% Penicillin/Streptomycin, 2 mM L-Glutamine	0.02% Versene
SK-N-FI cell line	Homo sapiens	Neuroblastoma	NA	Total RNA extraction	SK-N-FI	RPMI 1,640, 10% FBS, 1% Penicillin/Streptomycin, 2 mM L-Glutamine	0.02% Versene
SK-N-SH cell line	Homo sapiens	Neuroblastoma	NA	Total RNA extraction	SK-N-SH	RPMI 1,640, 10% FBS, 1% Penicillin/Streptomycin, 2 mM L-Glutamine	0.05% Trypsin/EDTA
SMS-KAN cell line	Homo sapiens	Neuroblastoma	NA	Total RNA extraction	SMS-KAN	RPMI 1,640, 10% FBS, 1% Penicillin/Streptomycin, 2 mM L-Glutamine	0.02% Versene
SMS-SAN cell line	Homo sapiens	Neuroblastoma	NA	Total RNA extraction	SMS-SAN	RPMI 1,640, 10% FBS, 1% Penicillin/Streptomycin, 2 mM L-Glutamine	0.02% Versene
Human Fetal Brain	Homo sapiens	Brain	NA	NA	Brain	NA	NA
Listed are the cell lines used in this study, matched PDX if applicable, and culturing media.							

**Table 2:** Associated metadata for the RNA-Seq assay

**Cell Line**	**Data ID**	**Assay Name**	**Raw Data File Name 1**	**Raw Data File Name 2**	**Data Repository**	**Data Accession Number**	**Derived Data File Name**	**Data Repository**	**Data Accession Number**
CHP-134	CHP134	RNA-Seq	CHP134.R1.fq	CHP134.R2.fq	GEO	GSM2371222	GSE89413_2016-10-30-NBL-cell-line-STAR-fpkm.txt.gz	GEO	GSE89413
CHP-212	CHP212	RNA-Seq	CHP212.R1.fq	CHP212.R2.fq	GEO	GSM2371223	GSE89413_2016-10-30-NBL-cell-line-STAR-fpkm.txt.gz	GEO	GSE89413
COG-N-415	COGN415	RNA-Seq	COGN415.R1.fq	COGN415.R2.fq	GEO	GSM2371224	GSE89413_2016-10-30-NBL-cell-line-STAR-fpkm.txt.gz	GEO	GSE89413
COG-N-440	COGN440	RNA-Seq	COGN440.R1.fq	COGN440.R2.fq	GEO	GSM2371225	GSE89413_2016-10-30-NBL-cell-line-STAR-fpkm.txt.gz	GEO	GSE89413
COG-N-453	COGN453	RNA-Seq	COGN453.R1.fq	COGN453.R2.fq	GEO	GSM2371226	GSE89413_2016-10-30-NBL-cell-line-STAR-fpkm.txt.gz	GEO	GSE89413
COG-N-471nb	COGN471	RNA-Seq	COGN471.R1.fq	COGN471.R2.fq	GEO	GSM2371227	GSE89413_2016-10-30-NBL-cell-line-STAR-fpkm.txt.gz	GEO	GSE89413
COG-N-496	COGN496	RNA-Seq	COGN496.R1.fq	COGN496.R2.fq	GEO	GSM2371228	GSE89413_2016-10-30-NBL-cell-line-STAR-fpkm.txt.gz	GEO	GSE89413
COG-N-519	COGN519	RNA-Seq	COGN519.R1.fq	COGN519.R2.fq	GEO	GSM2371229	GSE89413_2016-10-30-NBL-cell-line-STAR-fpkm.txt.gz	GEO	GSE89413
COG-N-534	COGN534	RNA-Seq	COGN534.R1.fq	COGN534.R2.fq	GEO	GSM2371230	GSE89413_2016-10-30-NBL-cell-line-STAR-fpkm.txt.gz	GEO	GSE89413
COG-N-549	COGN549	RNA-Seq	COGN549.R1.fq	COGN549.R2.fq	GEO	GSM2371231	GSE89413_2016-10-30-NBL-cell-line-STAR-fpkm.txt.gz	GEO	GSE89413
COG-N-557nb	COGN557	RNA-Seq	COGN557.R1.fq	COGN557.R2.fq	GEO	GSM2371232	GSE89413_2016-10-30-NBL-cell-line-STAR-fpkm.txt.gz	GEO	GSE89413
COG-N-561	COGN561	RNA-Seq	COGN561.R1.fq	COGN561.R2.fq	GEO	GSM2371233	GSE89413_2016-10-30-NBL-cell-line-STAR-fpkm.txt.gz	GEO	GSE89413
COG-N-573	COGN573	RNA-Seq	COGN573.R1.fq	COGN573.R2.fq	GEO	GSM2371234	GSE89413_2016-10-30-NBL-cell-line-STAR-fpkm.txt.gz	GEO	GSE89413
Felix (COG-N-426)	FELIX	RNA-Seq	FELIX.R1.fq	FELIX.R2.fq	GEO	GSM2371235	GSE89413_2016-10-30-NBL-cell-line-STAR-fpkm.txt.gz	GEO	GSE89413
IMR-05	IMR05	RNA-Seq	IMR05.R1.fq	IMR05.R2.fq	GEO	GSM2371236	GSE89413_2016-10-30-NBL-cell-line-STAR-fpkm.txt.gz	GEO	GSE89413
IMR-32	IMR32	RNA-Seq	IMR32.R1.fq	IMR32.R2.fq	GEO	GSM2371237	GSE89413_2016-10-30-NBL-cell-line-STAR-fpkm.txt.gz	GEO	GSE89413
KELLY	KELLY	RNA-Seq	KELLY.R1.fq	KELLY.R2.fq	GEO	GSM2371238	GSE89413_2016-10-30-NBL-cell-line-STAR-fpkm.txt.gz	GEO	GSE89413
LA-N-5	LAN5	RNA-Seq	LAN5.R1.fq	LAN5.R2.fq	GEO	GSM2371239	GSE89413_2016-10-30-NBL-cell-line-STAR-fpkm.txt.gz	GEO	GSE89413
LA-N-6	LAN6	RNA-Seq	LAN6.R1.fq	LAN6.R2.fq	GEO	GSM2371240	GSE89413_2016-10-30-NBL-cell-line-STAR-fpkm.txt.gz	GEO	GSE89413
NB-1	NB1	RNA-Seq	NB1.R1.fq	NB1.R2.fq	GEO	GSM2371241	GSE89413_2016-10-30-NBL-cell-line-STAR-fpkm.txt.gz	GEO	GSE89413
NB-16	NB16	RNA-Seq	NB16.R1.fq	NB16.R2.fq	GEO	GSM2371242	GSE89413_2016-10-30-NBL-cell-line-STAR-fpkm.txt.gz	GEO	GSE89413
NB-1643	NB1643	RNA-Seq	NB1643.R1.fq	NB1643.R2.fq	GEO	GSM2371243	GSE89413_2016-10-30-NBL-cell-line-STAR-fpkm.txt.gz	GEO	GSE89413
NB-1691	NB1691	RNA-Seq	NB1691.R1.fq	NB1691.R2.fq	GEO	GSM2371244	GSE89413_2016-10-30-NBL-cell-line-STAR-fpkm.txt.gz	GEO	GSE89413
NB-69	NB69	RNA-Seq	NB69.R1.fq	NB69.R2.fq	GEO	GSM2371245	GSE89413_2016-10-30-NBL-cell-line-STAR-fpkm.txt.gz	GEO	GSE89413
NB-EBc1	NBEBC1	RNA-Seq	NBEBC1.R1.fq	NBEBC1.R2.fq	GEO	GSM2371246	GSE89413_2016-10-30-NBL-cell-line-STAR-fpkm.txt.gz	GEO	GSE89413
NB-SD	NBSD	RNA-Seq	NBSD.R1.fq	NBSD.R2.fq	GEO	GSM2371247	GSE89413_2016-10-30-NBL-cell-line-STAR-fpkm.txt.gz	GEO	GSE89413
NBL-S	NBLS	RNA-Seq	NBLS.R1.fq	NBLS.R2.fq	GEO	GSM2371248	GSE89413_2016-10-30-NBL-cell-line-STAR-fpkm.txt.gz	GEO	GSE89413
NGP	NGP	RNA-Seq	NGP.R1.fq	NGP.R2.fq	GEO	GSM2371249	GSE89413_2016-10-30-NBL-cell-line-STAR-fpkm.txt.gz	GEO	GSE89413
NLF	NLF	RNA-Seq	NLF.R1.fq	NLF.R2.fq	GEO	GSM2371250	GSE89413_2016-10-30-NBL-cell-line-STAR-fpkm.txt.gz	GEO	GSE89413
NMB	NMB	RNA-Seq	NMB.R1.fq	NMB.R2.fq	GEO	GSM2371251	GSE89413_2016-10-30-NBL-cell-line-STAR-fpkm.txt.gz	GEO	GSE89413
RPE-1	RPE1	RNA-Seq	RPE1.R1.fq	RPE1.R2.fq	GEO	GSM2371252	GSE89413_2016-10-30-NBL-cell-line-STAR-fpkm.txt.gz	GEO	GSE89413
SH-SY5Y	SHSY5Y	RNA-Seq	SHSY5Y.R1.fq	SHSY5Y.R2.fq	GEO	GSM2371253	GSE89413_2016-10-30-NBL-cell-line-STAR-fpkm.txt.gz	GEO	GSE89413
SK-N-AS	SKNAS	RNA-Seq	SKNAS.R1.fq	SKNAS.R2.fq	GEO	GSM2371254	GSE89413_2016-10-30-NBL-cell-line-STAR-fpkm.txt.gz	GEO	GSE89413
SK-N-BE(2)	SKNBE2	RNA-Seq	SKNBE2.R1.fq	SKNBE2.R2.fq	GEO	GSM2371255	GSE89413_2016-10-30-NBL-cell-line-STAR-fpkm.txt.gz	GEO	GSE89413
SK-N-BE(2)-C	SKNBE2C	RNA-Seq	SKNBE2C.R1.fq	SKNBE2C.R2.fq	GEO	GSM2371256	GSE89413_2016-10-30-NBL-cell-line-STAR-fpkm.txt.gz	GEO	GSE89413
SK-N-DZ	SKNDZ	RNA-Seq	SKNDZ.R1.fq	SKNDZ.R2.fq	GEO	GSM2371257	GSE89413_2016-10-30-NBL-cell-line-STAR-fpkm.txt.gz	GEO	GSE89413
SK-N-FI	SKNFI	RNA-Seq	SKNFI.R1.fq	SKNFI.R2.fq	GEO	GSM2371258	GSE89413_2016-10-30-NBL-cell-line-STAR-fpkm.txt.gz	GEO	GSE89413
SK-N-SH	SKNSH	RNA-Seq	SKNSH.R1.fq	SKNSH.R2.fq	GEO	GSM2371259	GSE89413_2016-10-30-NBL-cell-line-STAR-fpkm.txt.gz	GEO	GSE89413
SMS-KAN	SMSKAN	RNA-Seq	SMSKAN.R1.fq	SMSKAN.R2.fq	GEO	GSM2371260	GSE89413_2016-10-30-NBL-cell-line-STAR-fpkm.txt.gz	GEO	GSE89413
SMS-SAN	SMSSAN	RNA-Seq	SMSSAN.R1.fq	SMSSAN.R2.fq	GEO	GSM2371261	GSE89413_2016-10-30-NBL-cell-line-STAR-fpkm.txt.gz	GEO	GSE89413
Human Fetal Brain	HU-FETAL-BRAIN	RNA-Seq	HU-FETAL-BRAIN.R1.fq	HU-FETAL-BRAIN.R2.fq	GEO	GSM2371262	GSE89413_2016-10-30-NBL-cell-line-STAR-fpkm.txt.gz	GEO	GSE89413
Listed are the RNA sequencing files available for each cell line, along with GEO accession numbers.									

**Table 3:** Genetic lesion profiles for the neuroblastoma cell lines

**Cell Line**	**MYCN status**	**1p36 del**	**3p26 del**	**11q23 del**	**17q21-qter unbal gain**	**ALK mutation**	**p53 mutation**
CHP-134	Amplified	LOH p32.3-pter; Gain p34.3-p36.22; Loss p36.22-pter	Gain/AI p26.3	None	Gain q12-qter	WT	WT
CHP-212	Amplified	Loss p13.2-pter	Gain/AI p26.3	cnLOH 23.3	Gain q12-qter	WT	WT
COG-N-415	Amplified	Unknown	Unknown	Unknown	Unknown	F1174L	WT
COG-N-440	Amplified	Unknown	Unknown	Unknown	Unknown	WT	WT
COG-N-453	Amplified	Unknown	Unknown	Unknown	Unknown	F1174L	WT
COG-N-471nb	Amplified	Unknown	Unknown	Unknown	Unknown	WT	WT
COG-N-496	Amplified	Unknown	Unknown	Unknown	Unknown	Unknown	Unknown
COG-N-519	Amplified	Unknown	Unknown	Unknown	Unknown	Unknown	Unknown
COG-N-534	Non-amplified	Unknown	Unknown	Unknown	Unknown	Unknown	Unknown
COG-N-549	Non-amplified	Unknown	Unknown	Unknown	Unknown	Unknown	Unknown
COG-N-557nb	Amplified	Unknown	Unknown	Unknown	Unknown	Unknown	Unknown
COG-N-561	Amplified	Unknown	Unknown	Unknown	Unknown	Unknown	Unknown
COG-N-573	Amplified	Unknown	Unknown	Unknown	Unknown	Unknown	Unknown
FELIX (COG-N-426)	Non-amplified	Unknown	Unknown	Unknown	Unknown	F1245C	WT
IMR-05	Amplified	Gain+LOH p32.3-pter	Gain/AI p24.3-26.3	Loss q22.1-qter	Gain q21.2-qter	WT	WT
IMR-32	Amplified	Loss p32.3-pter	Loss p12.3	cnLOH q23.1	Gain q21.2-qter	WT	WT
KELLY	Amplified	LOH p21.3-pter; Loss p36.32; Gain p36.33	Loss p26.2	Loss q23.3-qter	Gain q21.2-qter	F1174L	P177T
LA-N-5	Amplified	Loss p33-pter	None	None	Gain q21.2-qter	R1275Q	WT
LA-N-6	Non-amplified	p36.12-pter; cnLOH p35.3-p36.11	Loss p14.3-pter	Loss q13.4-qter	Gain q12-qter	WT	WT
NB-1	Amplified	Loss p32.2-pter	Gain p24.1-pter	cnLOH q23.1	Gain q22-qter	WT; amplified	WT
NB-16	Non-amplified	None	None	None	Unresolvable	WT	R248W, R175H
NB-1,643	Amplified	Loss p34.2-pter	None	Loss q23.1-qter	Gain q21.31-qter	R1275Q	WT
NB-1,691	Amplified	None	Loss p26.1	LOH q11-qter; Loss q22.1-q24.3	None?	WT	WT
NB-69	Non-amplified	Loss p13.3-pter	None	cnLOH q23.1, q23.3	Gain q12-qter	WT	WT
NB-EBc1	Non-amplified	Loss p35.2-pter	Gain p26.1-pter	Loss q21-24.2	Gain q21.31-qter	WT	WT
NB-SD	Amplified	Loss p21.3-pter	Loss p13-pter	cnLOH q23.2-23.3	Gain q12-qter	F1174L	C176F
NBL-S	Non-amplified	None	None	Loss q14.1-qter	None	WT	WT
NGP	Amplified	cnLOH p32.3-pter	Gain p25.3-pter	Loss q22.1-qter	Gain q21.1-qter	WT	A159D, C141W
NLF	Amplified	Loss p32.2-pter	Loss/AI p26.1; AI p26.2; Gain 26.3	Loss/AI q23.3-qter	Gain q21.2-qter	WT	V203M
NMB	Amplified	cnLOH p34.2-pter	None	Loss q21-qter	LOH/Gain q11.2-qter	WT	G245S
RPE-1	Non-amplified	Unknown	Unknown	Unknown	Unknown	WT	WT
SH-SY5Y	Non-amplified	None	None	Loss q22.1-q24.2	Gain q21.31-qter	F1174L	WT
SK-N-AS	Non-amplified	Loss p36.22-36.32	Loss p14.2-pter	q13.4-qter	Gain q21.31-qter	WT	H168R
SK-N-BE(2)	Amplified	cnLOH p21.3-pter	Loss p14.2-pter	Gain/AI q13.1-qter	Gain q12-qter	WT	C135F
SK-N-BE(2)-C	Amplified	cnLOH p21.3-pter	Loss p14.2-pter	LOH q11-qter; Gain q13.4-qter	Gain q12-qter	WT	C135F
SK-N-DZ	Amplified	None	Gain/AI p26.1-pter	Loss q21-qter	Gain q21.2-qter	WT	R110L
SK-N-FI	Non-amplified	None	None	None	Gain q21.31-qter	WT	M246R
SK-N-SH	Non-amplified	None	None	None	Gain q21.31-qter	F1174L	WT
SMS-KAN	Amplified	Loss p13.3-pter	None	Loss q14.2-q23.3	Gain q11.1-qter	WT	WT
SMS-SAN	Amplified	Loss p32.3-pter	None	Loss q25	Gain q21.2-qter	F1174L	WT
Listed are collated data for common genetic lesions in neuroblastoma: *MYCN* amplification status, 1p^24^, 3p^25^, and 11q^26^ deletion status, 17q unbalanced gain^27^ status, *ALK* mutation status^18^, and TP53 mutation status^18^. Alterations for 1p, 3p, 11q, and 17q are reported from both visual inspection of SNP arrays in Illumina’s Genome Studio as well as processed files from Nexus Copy Number software (Biodiscovery, El Segundo, CA); see Data Citation 3 (del=deletion, cnLOH=copy neutral loss of heterozygosity, AI=allelic imbalance).							

## References

[d1] *Gene Expression Omnibus* Harenza J. Diamond M. A. Hart L. S. Maris J. M. 2016 GSE89413

[d2] *NBCI Bioproject* 2009 PRJNA89523

[d3] *Gene Expression Omnibus* Harenza J. Diamond M. A. Maris J. M. 2016 GSE89968

