# Corrigendum: Transcriptomic profiling of 39 commonly-used neuroblastoma cell lines

**DOI:** 10.1038/sdata.2017.183

**Published:** 2017-12-05

**Authors:** Jo Lynne Harenza, Maura A. Diamond, Rebecca N. Adams, Michael M. Song, Heather L. Davidson, Lori S. Hart, Maiah H. Dent, Paolo Fortina, C. Patrick Reynolds, John M. Maris

*Scientific Data* 4:170033 doi: 10.1038/sdata.2017.33 (2017); Published 28 March 2017; Updated: 5 December 2017.

The growth media for the cell lines CHP-212 and IMR-32 listed in Table 1 of this Data Descriptor were incorrectly stated.

- For the cell line CHP-212, the correct growth medium is: EMEM (with non-essential amino acids) and Ham′s F12 (1:1 mixture), 10% FBS, 1% Penicillin/Streptomycin, 2 mM L-Glutamine.
- For the cell line IMR-32, the correct growth medium is: EMEM (with non-essential amino acids), 10% FBS, 1% Penicillin/Streptomycin, 2 mM L-Glutamine.
- The COG cell lines were grown in antibiotic-free medium.

The cell line labelled as ‘NB-16’ in Fig. 2, Table 1, and Table 2 was incorrectly recorded. The cell line should be listed as the rhabdomyosarcoma cell line, RD. GEO Series GSE89969 has been updated to reflect this error. We subsequently re-performed every analysis in the paper following exclusion of NB-16 and none of the conclusions of the manuscript change. As expected, all cell line—patient correlations are slightly higher (e.g.,: Cell Line—Patient Log2Expression R with NB-16=0.824, Cell Line—Patient Log2Expression R without NB-16=0.831). All other cell lines have been properly STR-verified using www.atcc.org and www.cogcell.org, and are labelled correctly.

